# Supplementary material for: Weak localized electrons enhance electronic coherence for efficient photocatalytic uranium removal from nuclear wastewater
Source: Nat Commun. 2026 Feb 27;17:3262. doi: 10.1038/s41467-026-69178-6 (PMC13065756; doi:10.1038/s41467-026-69178-6)
Supplement: Supplementary file 1 — Supplementary Information file [file 41467_2026_69178_MOESM1_ESM.pdf]

Supplementary information

Weak Localized Electrons Enhance Electronic Coherence for Efficient Photocatalytic Uranium Removal from Nuclear Wastewater

Yachao Xu[1], Ruolan Zhao[2,3], Youxing Liu*[1,2], Ying Wang[3], Zheng Lin[1], Zongqiang Sun[1], Peng Yu[3], Mingchuan Luo[1], Shaojun Guo*[1]

[1]School of Materials Science and Engineering, Peking University, Beijing 100871, China.

[2]Beijing Key Laboratory of Electrochemical Process and Technology for Materials, Beijing University of Chemical Technology, Beijing 100029, China

[3]School of Physics and Electronic Engineering, Harbin Normal University, Harbin 150025, China.

E-mail: liuyxpost@pku.edu.cn; guosj@pku.edu.cn

Table of Contents

1. Supplementary Figure..3

2. Supplementary Table……..69

3. References...75

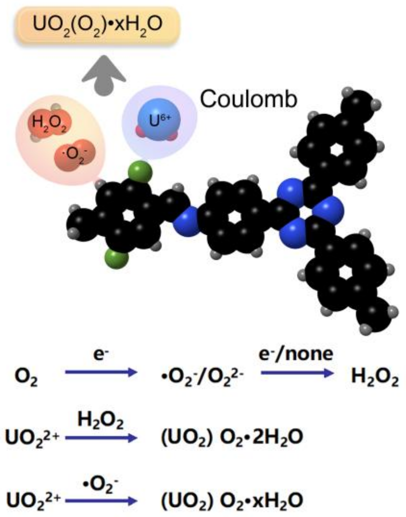

Supplementary Fig. 1 | The formation process of $(UO_2)O_2{\cdot}xH_2O$. Schematic illustration of the photocatalytic formation process of $(UO_2)O_2{\cdot}xH_2O$.

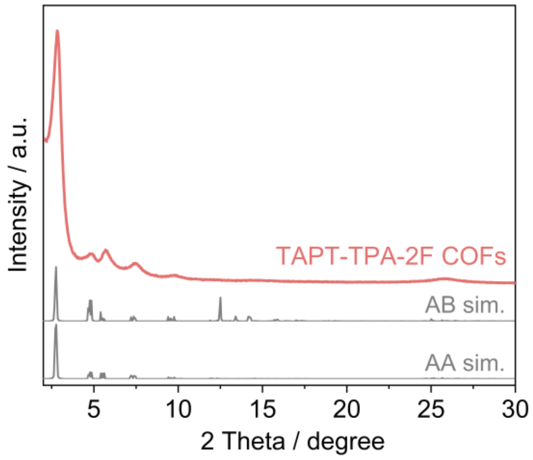

Supplementary Fig. 2 | Crystal structure characterization. Powder X-ray diffraction (XRD) pattern of TAPT-TPA-2F COFs.

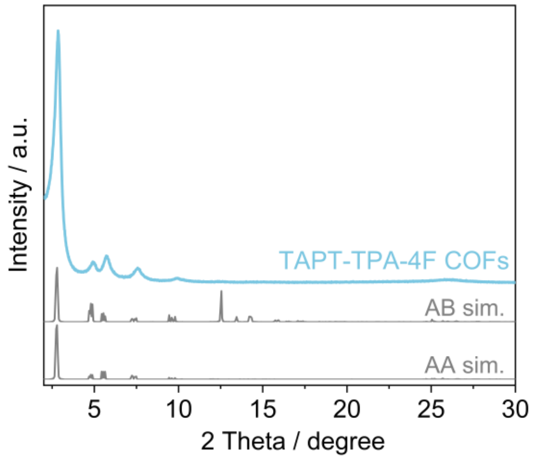

Supplementary Fig. 3 | Crystal structure characterization. XRD pattern of TAPT-TPA-4F COFs.

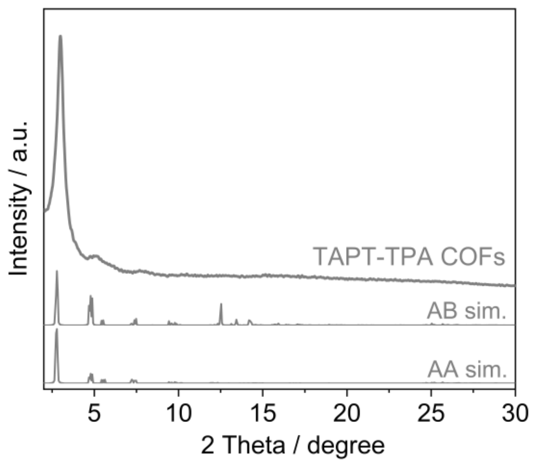

Supplementary Fig. 4 | Crystal structure characterization. XRD pattern of TAPT-TPA COFs.

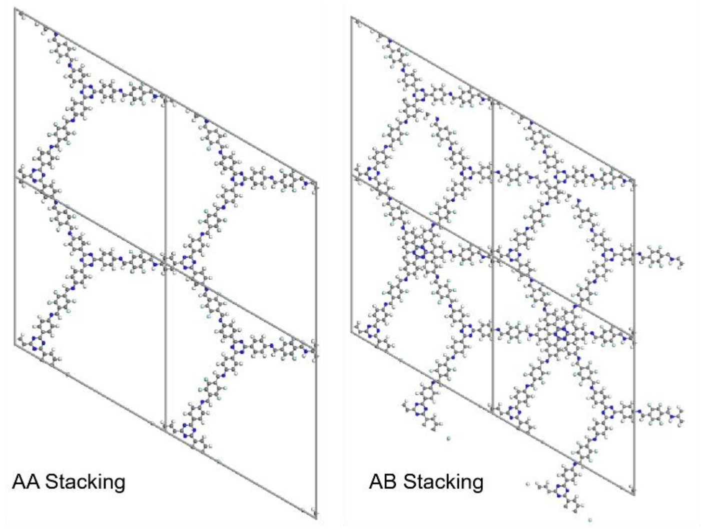

Supplementary Fig. 5 | Simulated molecular structures. Illustrations of AA and AB stacking configurations of TAPT-TPA-4F COFs obtained from molecular structure simulations.

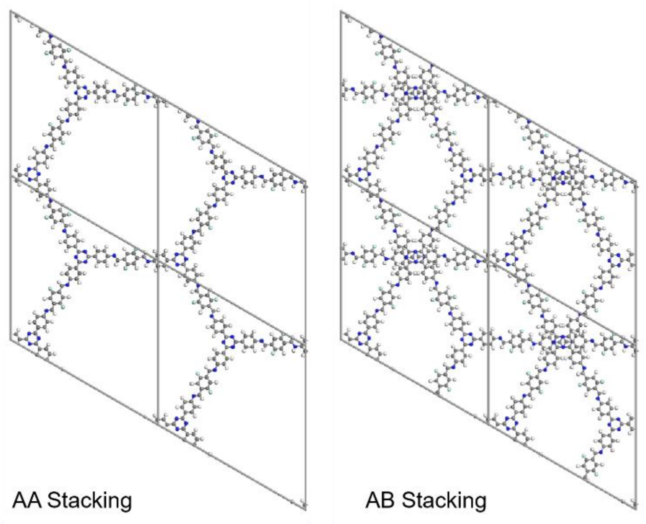

Supplementary Fig. 6 | Simulated molecular structures. Illustrations of AA and AB stacking configurations of TAPT-TPA-2F COFs obtained from molecular structure simulations.

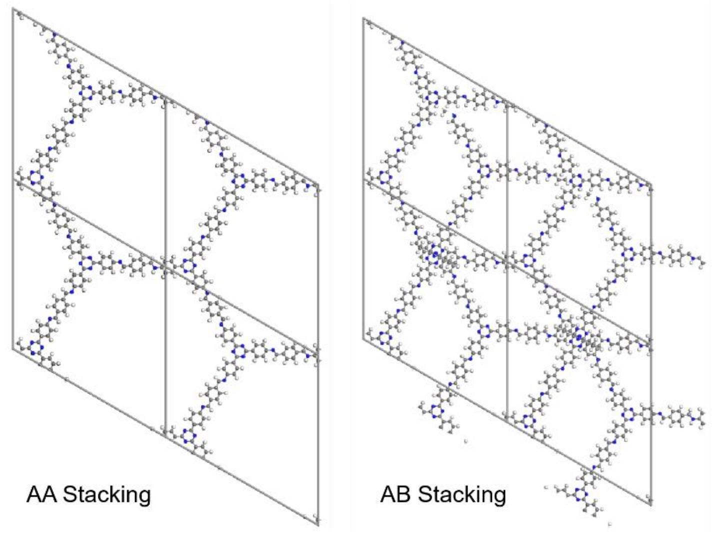

Supplementary Fig. 7 | Simulated molecular structures. Illustrations of AA and AB stacking configurations of TAPT-TPA COFs obtained from molecular structure simulations.

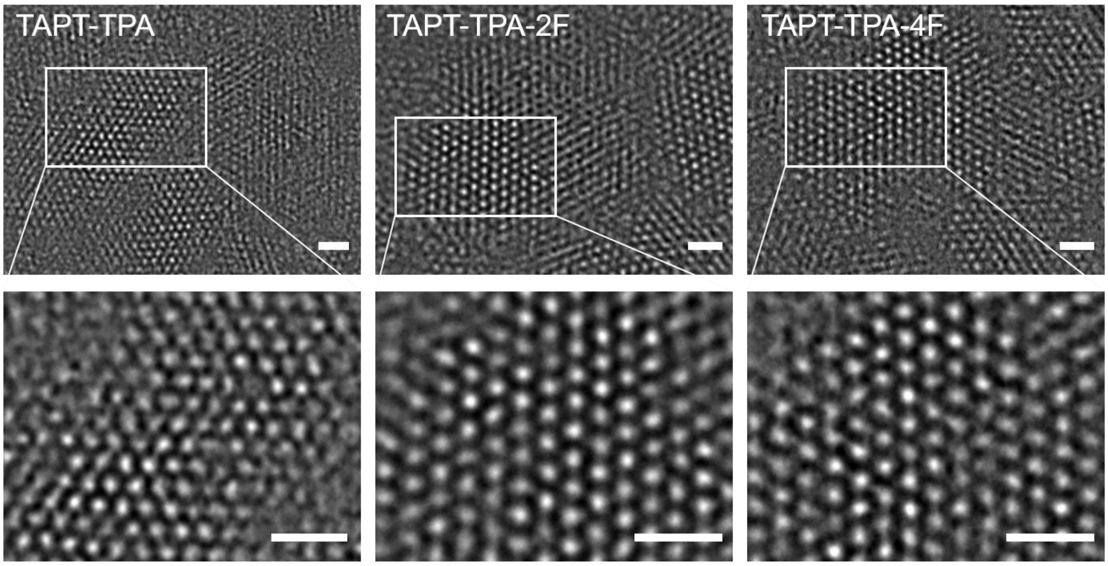

Supplementary Fig. 8 | Molecular microscopic characterization. High-resolution TEM images of TAPT-TPA, TAPT-TPA-2F and TAPT-TPA-4F, scale bar: 10 nm.

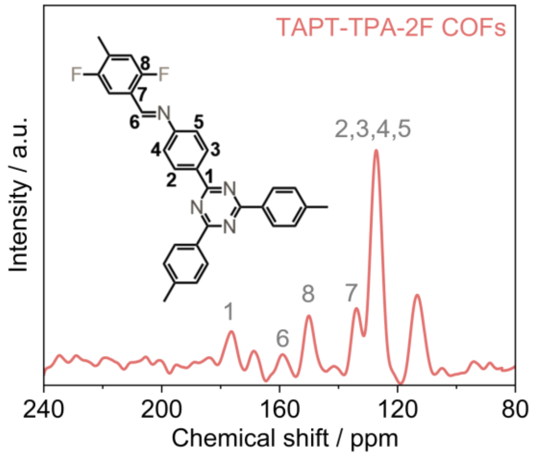

Supplementary Fig. 9 | Chemical structure characterization. Solid-state ^{13}C NMR spectrum of TAPT-TPA-2F COFs.

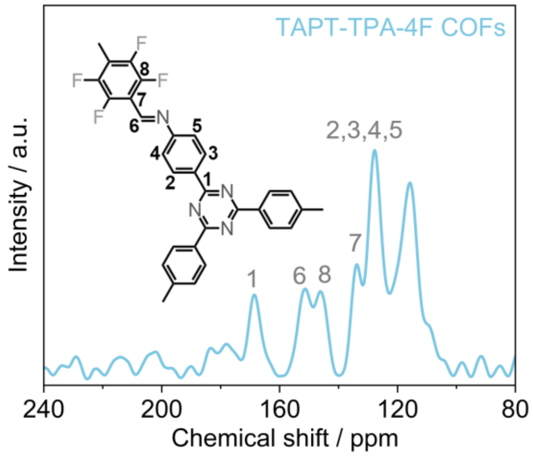

Supplementary Fig. 10 | Chemical structure characterization. Solid-state ^{13}C NMR spectrum of TAPT-TPA-4F COFs.

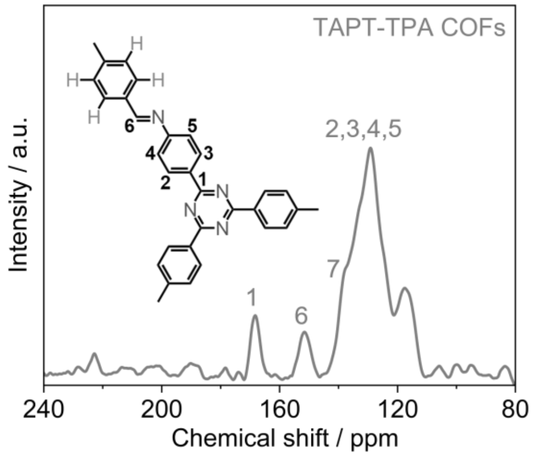

Supplementary Fig. 11 | Chemical structure characterization. Solid-state ^{13}C NMR spectrum of TAPT-TPA COFs.

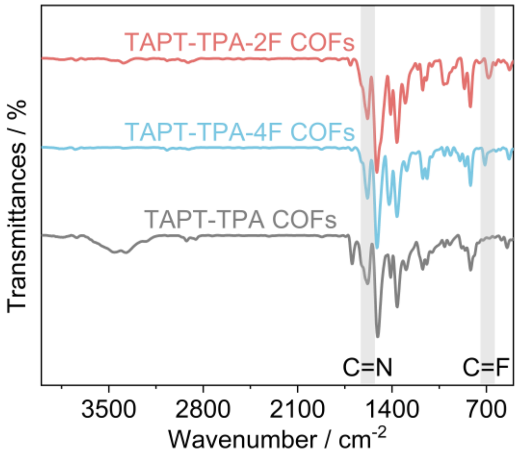

Supplementary Fig. 12 | Chemical bond characterization. FT-IR transmittance spectra of TAPT-TPA-2F COFs, TAPT-TPA-4F COFs and TAPT-TPA COFs.

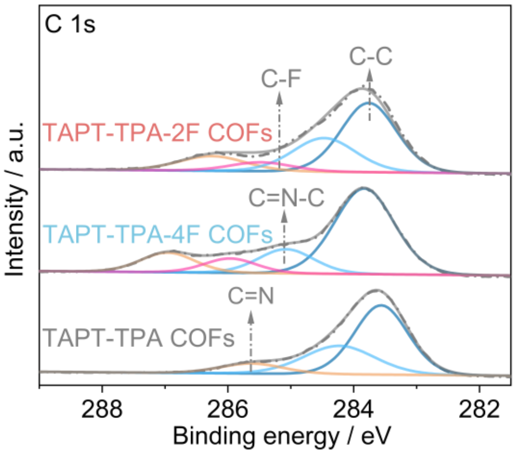

Supplementary Fig. 13 | Chemical bond characterization. High-resolution C 1s XPS spectra of TAPT-TPA-2F COFs, TAPT-TPA-4F COFs and TAPT-TPA COFs.

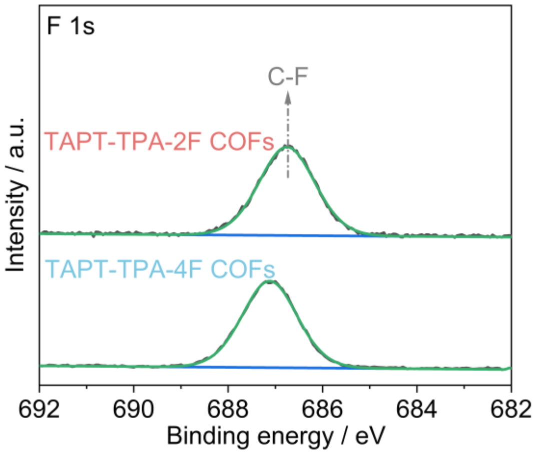

Supplementary Fig. 14 | Chemical bond characterization. High-resolution F 1s XPS spectra of TAPT-TPA-2F and TAPT-TPA-4F COFs.

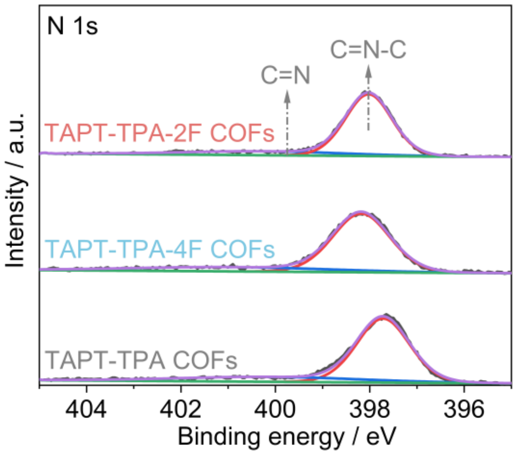

Supplementary Fig. 15 | Chemical bond characterization. High-resolution N 1s XPS spectra of TAPT-TPA-2F, TAPT-TPA-4F and TAPT-TPA COFs.

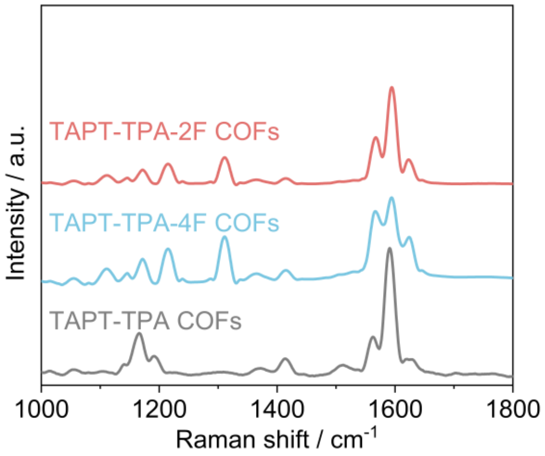

Supplementary Fig. 16 | Chemical bond characterization. Raman spectra of TAPT-TPA-2F, TAPT-TPA-4F and TAPT-TPA COFs.

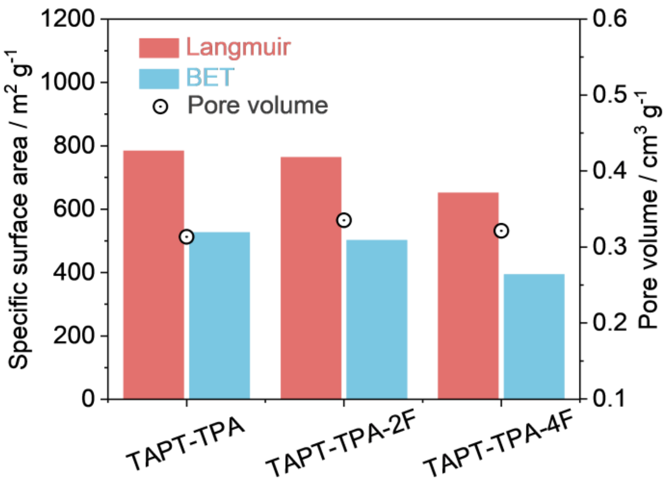

Supplementary Fig. 17 | Molecular structural characterization. The surface area and pore volume of TAPT-TPA(N^+) COFs and TAPT-TPA COFs.

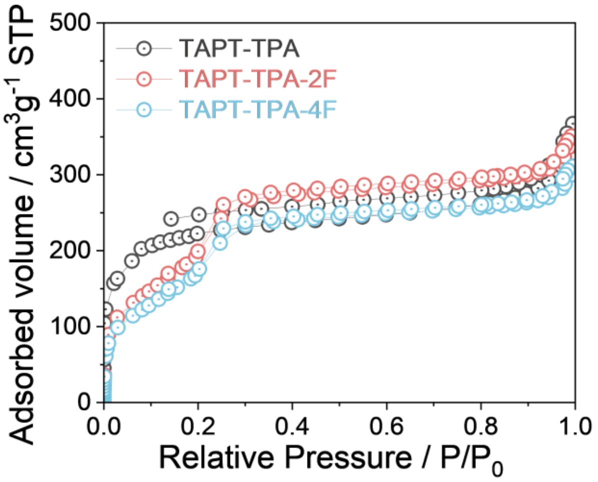

Supplementary Fig. 18 | Molecular structural characterization. N_2 adsorption-desorption isotherms of TAPT-TPA, TAPT-TPA–2F and TAPT-TPA-4F.

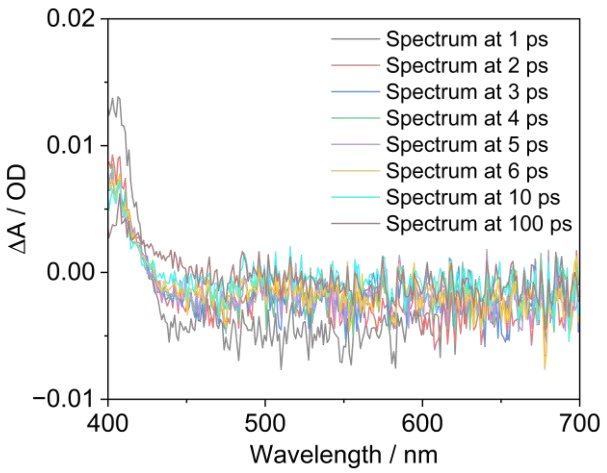

Supplementary Fig. 19 | Femtosecond transient absorption spectra. Transient absorption (TA) signals of TAPT-TPA-2F COFs recorded on the femtosecond to nanosecond timescales.

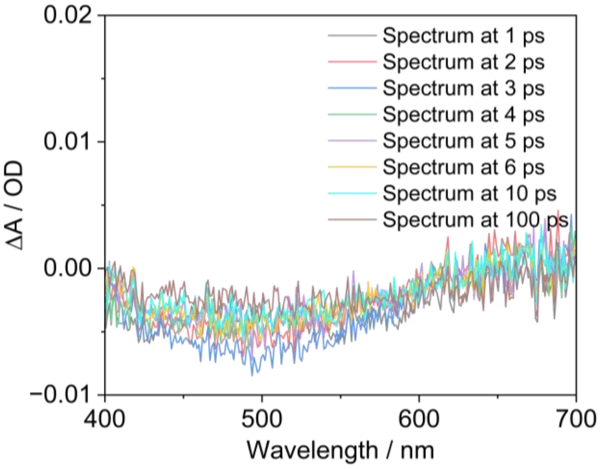

Supplementary Fig. 20 | Femtosecond transient absorption spectra. TA signals of TAPT-TPA-4F COFs measured on the femtosecond to nanosecond timescales.

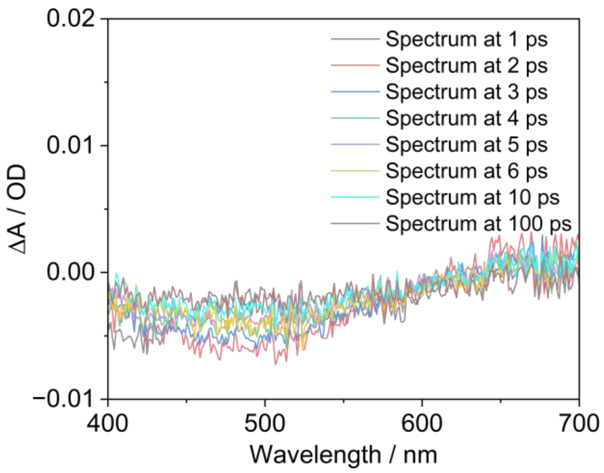

Supplementary Fig. 21 | Femtosecond transient absorption spectra. TA signals of TAPT-TPA COFs measured on the femtosecond to nanosecond timescales.

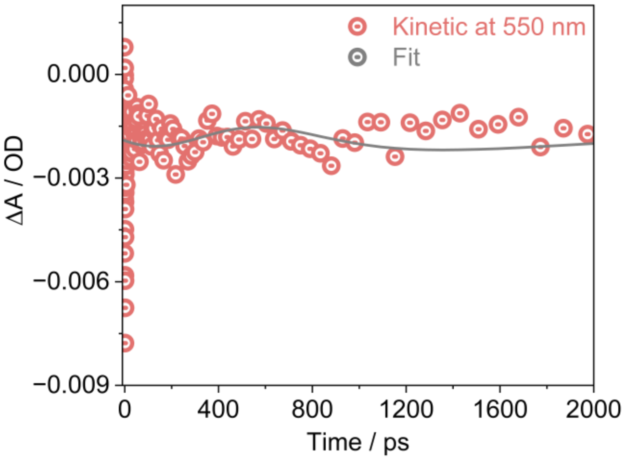

Supplementary Fig. 22 | Kinetic trace and exponential fitting. Femtosecond transient absorption kinetic profile of TAPT-TPA-2F COFs probed at 550 nm, showing experimental data (red circles) and exponential fitting curve (gray line). The decay dynamics reveal the photogenerated carrier relaxation behavior on the picosecond timescale.

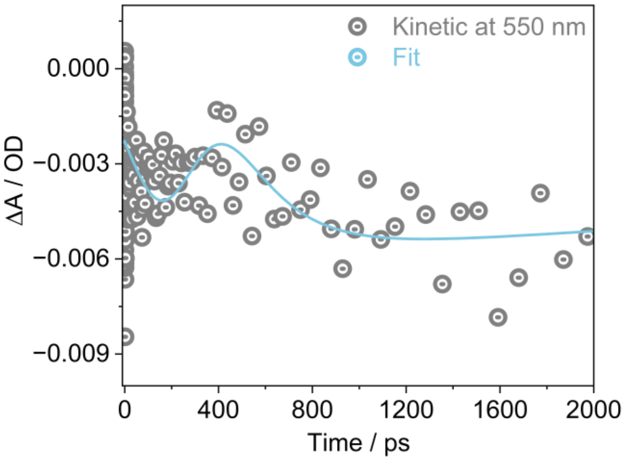

Supplementary Fig. 23 | Kinetic trace and exponential fitting. Femtosecond transient absorption kinetic profile of TAPT-TPA-4F COFs probed at 550 nm, showing experimental data (red circles) and exponential fitting curve (gray line). The decay dynamics reveal the photogenerated carrier relaxation behavior on the picosecond timescale.

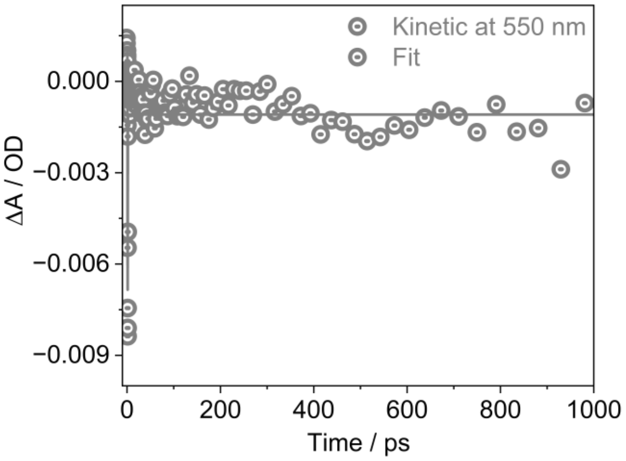

Supplementary Fig. 24 | Kinetic trace and exponential fitting. Femtosecond transient absorption kinetic profile of TAPT-TPA COFs probed at 550 nm, showing experimental data (red circles) and exponential fitting curve (gray line). The decay dynamics reveal the photogenerated carrier relaxation behavior on the picosecond timescale.

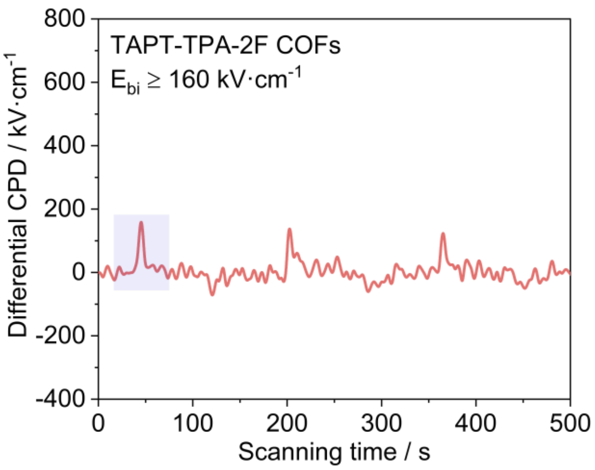

Supplementary Fig. 25 | Differential surface photovoltage profiling. Differential contact potential difference (CPD) profiles of TAPT-TPA-2F COFs under continuous light illumination, measured by surface photovoltage microscopy.

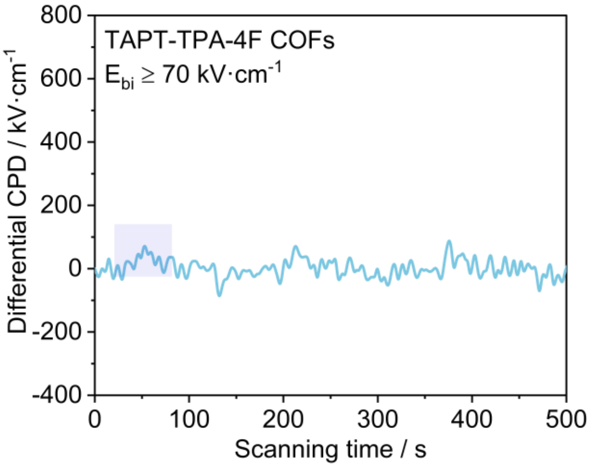

Supplementary Fig. 26 | Differential surface photovoltage profiling. Differential CPD profiles of TAPT-TPA-4F COFs under continuous light illumination, measured by surface photovoltage microscopy.

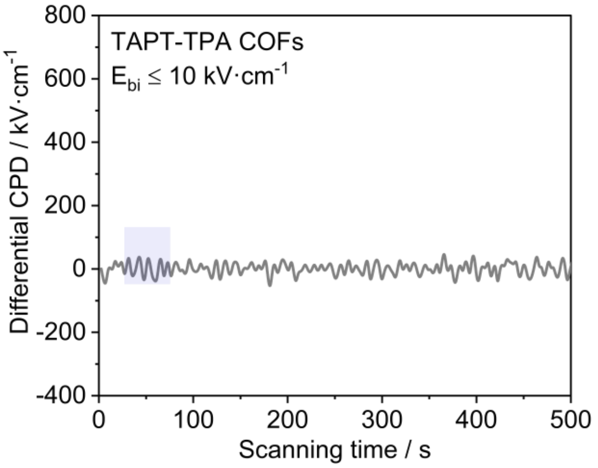

Supplementary Fig. 27 | Differential surface photovoltage profiling. Differential CPD profiles of TAPT-TPA COFs under continuous light illumination, measured by surface photovoltage microscopy.

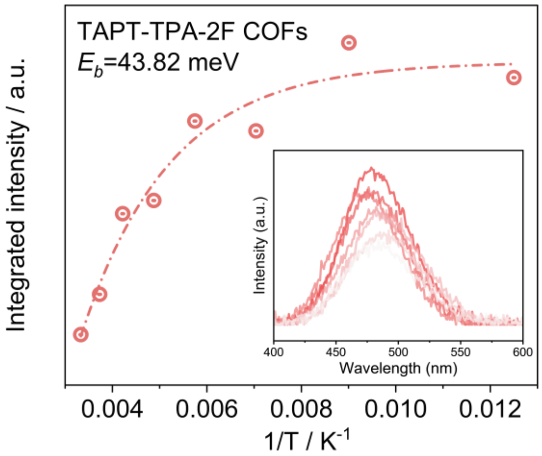

Supplementary Fig. 28 | Temperature-dependent photoluminescence spectra. PL spectra of TAPT-TPA-2F COFs recorded in the temperature range of 80–300 K.

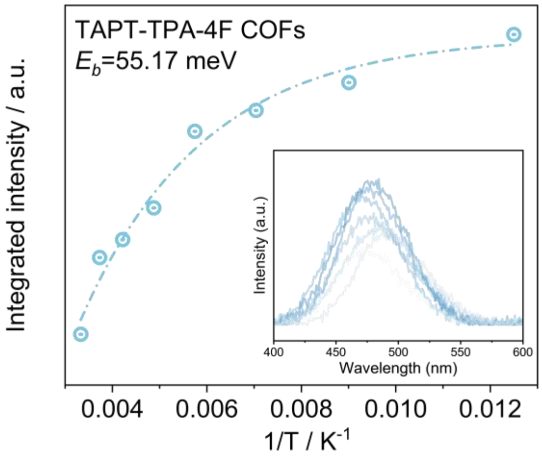

Supplementary Fig. 29 | Temperature-dependent photoluminescence spectra. PL spectra of TAPT-TPA-4F COFs recorded in the temperature range of 80–300 K.

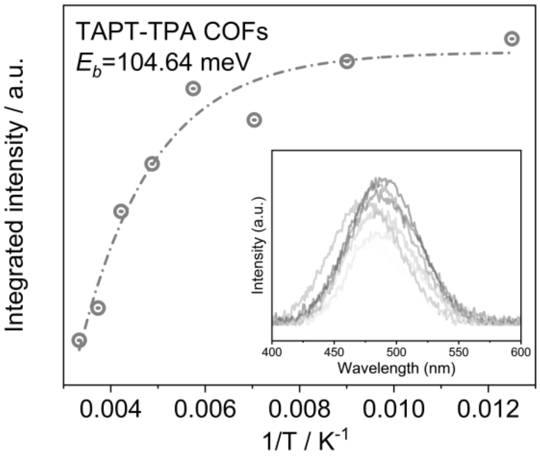

Supplementary Fig. 30 | Temperature-dependent photoluminescence spectra. PL spectra of TAPT-TPA COFs recorded in the temperature range of 80–300 K.

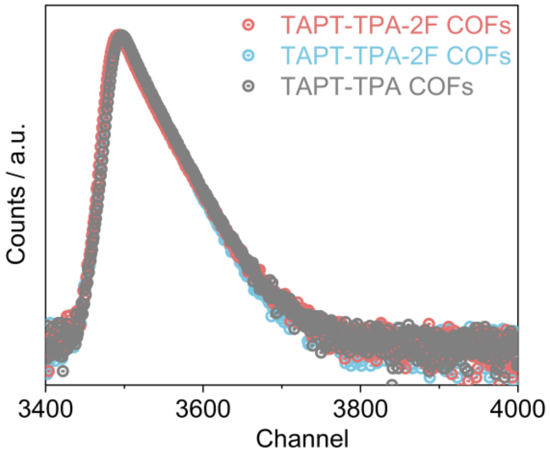

Supplementary Fig. 31 | Positron annihilation lifetime spectroscopy. Positron annihilation lifetime spectra of TAPT-TPA, TAPT-TPA-2F, and TAPT-TPA-4F COFs.

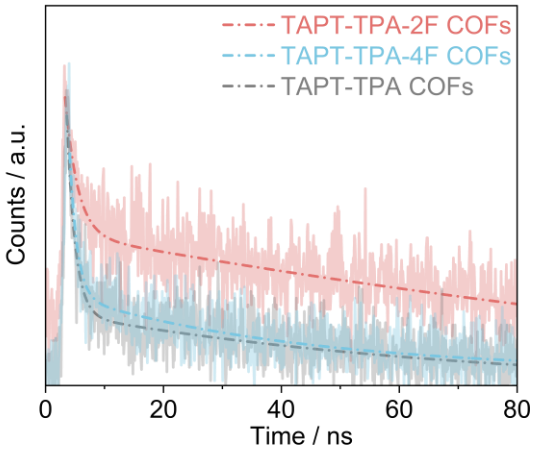

Supplementary Fig. 32 | Time-resolved photoluminescence spectra. Time-resolved fluorescence spectra of TAPT-TPA-2F, TAPT-TPA-4F and TAPT-TPA COFs.

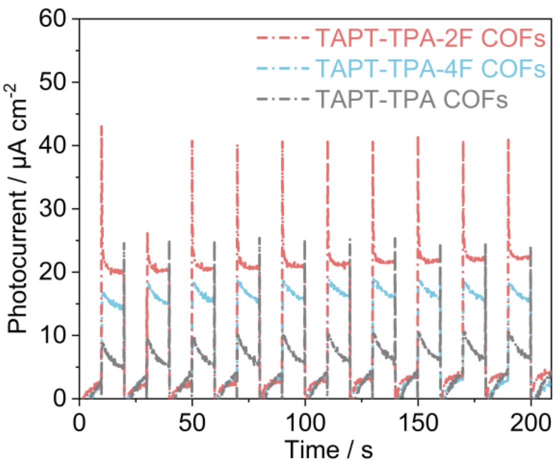

Supplementary Fig. 33 | Transient photocurrent response. Photoelectrochemical current–time (I–t) curves of TAPT-TPA-2F, TAPT-TPA-4F and TAPT-TPA COFs under chopped light irradiation.

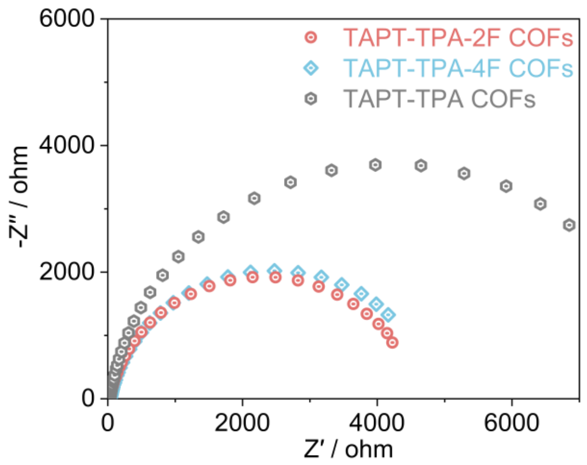

Supplementary Fig. 34 | Electrochemical impedance spectroscopy. Nyquist plots of TAPT-TPA-2F, TAPT-TPA-4F and TAPT-TPA COFs obtained from electrochemical impedance spectroscopy (EIS).

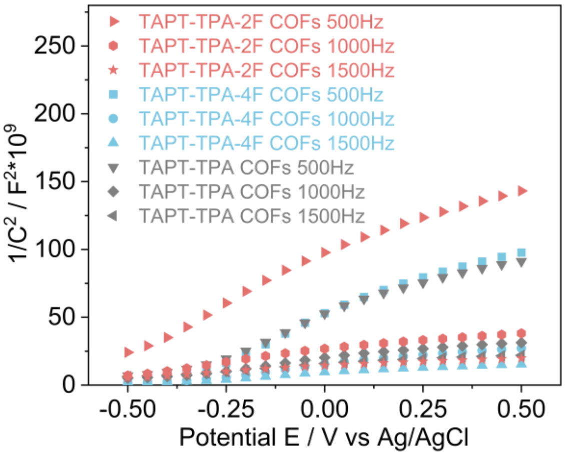

Supplementary Fig. 35 | Mott–Schottky analysis. Mott–Schottky plots of TAPT-TPA-2F, TAPT-TPA-4F and TAPT-TPA COFs.

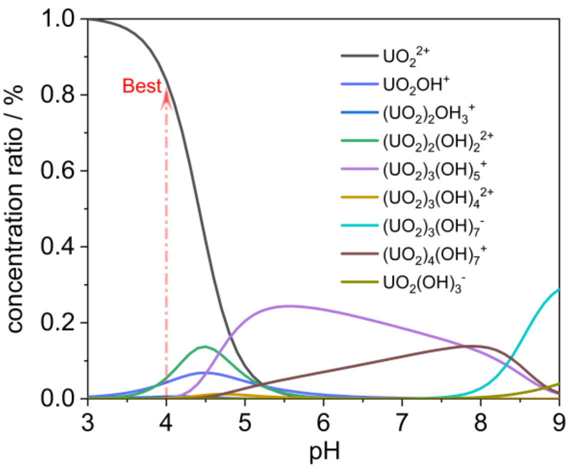

Supplementary Fig. 36 | Simulated speciation of uranyl ions at different pH values. Distribution of uranyl species as a function of pH, illustrating the predominant coordination forms and hydrolysis/complexation equilibria under varying acidic and alkaline conditions.

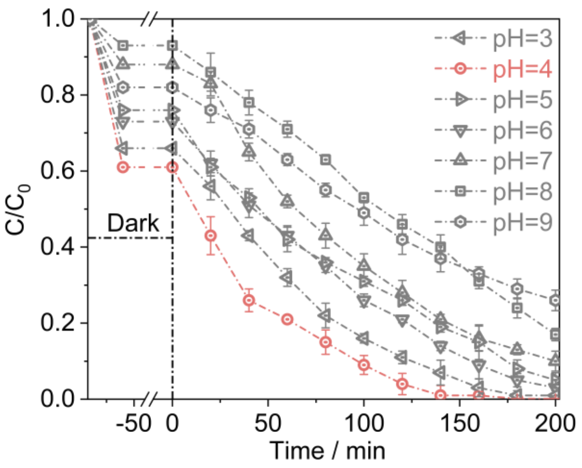

Supplementary Fig. 37 | Photocatalytic U(VI) removal performance under different pH conditions. U(VI) removal efficiencies of TAPT-TPA-2F, TAPT-TPA-4F and TAPT-TPA COFs evaluated across a range of pH values (T = 298K, UO_2^{2+} = 100 ppm, pH = 3-9). Data are presented as mean values ± SD (n = 3).

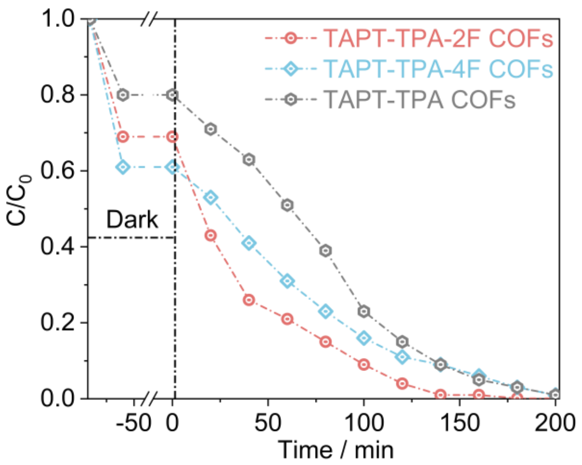

Supplementary Fig. 38 | U(VI) removal efficiencies. Removal efficiencies of U(VI) using TAPT-TPA-2F, TAPT-TPA-4F and TAPT-TPA COFs (T = 298K, UO_2^{2+} = 100ppm, pH = 4).

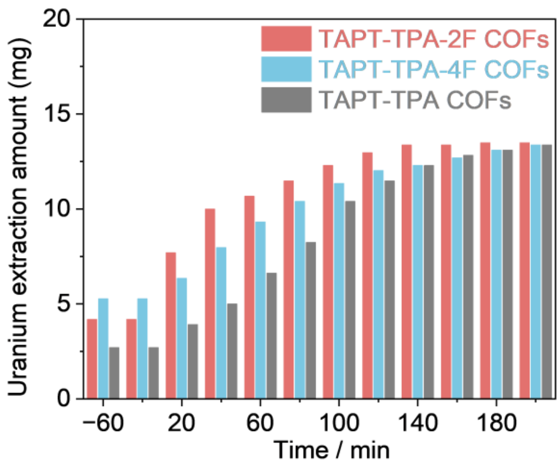

Supplementary Fig. 39 | Uranium extraction amount over time. Time-dependent uranium extraction performance of TAPT–TPA–2F, TAPT–TPA–4F, and TAPT–TPA COFs.

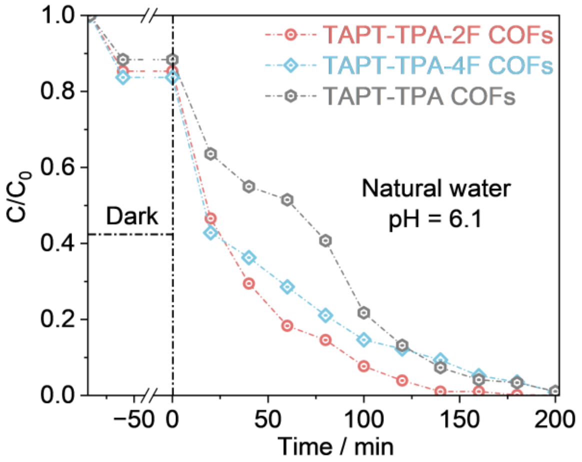

Supplementary Fig. 40 | Uranium removal efficiency in natural water over time. Time-dependent uranium removal performance of TAPT–TPA–2F, TAPT–TPA–4F, and TAPT–TPA COFs under dark and light conditions.

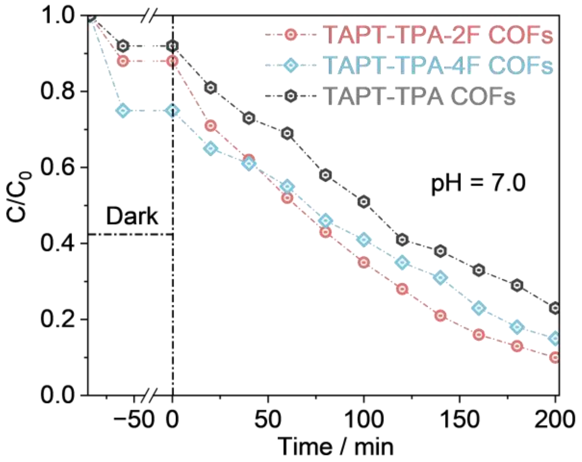

Supplementary Fig. 41 | Uranium removal efficiency at pH = 7. Time-dependent uranium removal performance of TAPT–TPA–2F, TAPT–TPA–4F, and TAPT–TPA COFs under neutral conditions.

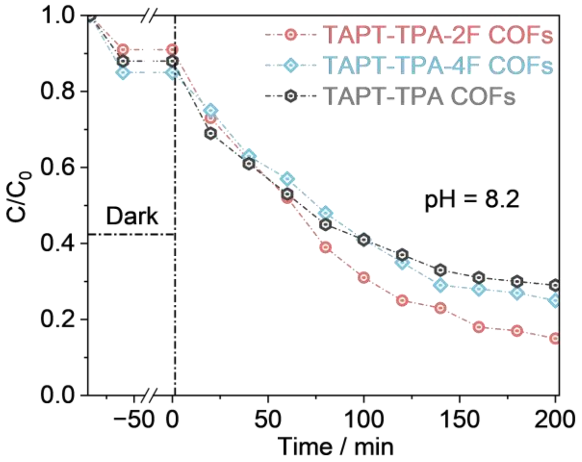

Supplementary Fig. 42 | Uranium removal efficiency at pH = 8.2. Time-dependent uranium removal performance of TAPT–TPA–2F, TAPT–TPA–4F, and TAPT–TPA COFs under neutral conditions.

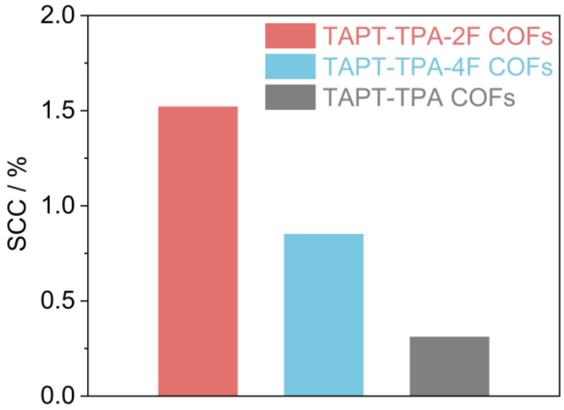

Supplementary Fig. 43 | Solar-to-chemical conversion efficiencies. Comparison of solar-to-chemical conversion (SCC) efficiencies for TAPT-TPA-2F, TAPT-TPA-4F and TAPT-TPA COFs under simulated sunlight irradiation.

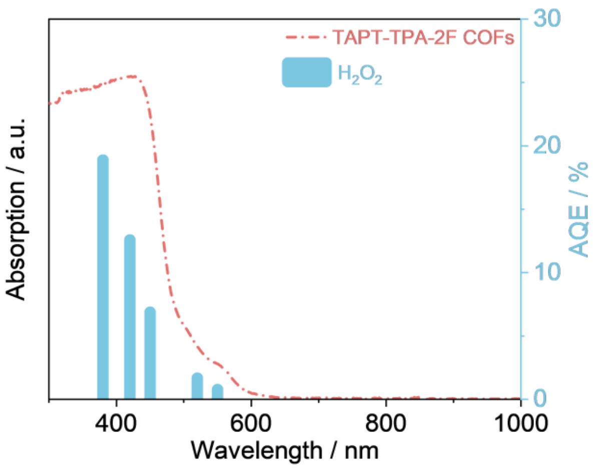

Supplementary Fig. 44 | Apparent quantum efficiency (AQE) and optical absorption spectrum. Wavelength-dependent AQE and corresponding absorption spectrum of TAPT–TPA–2F COFs.

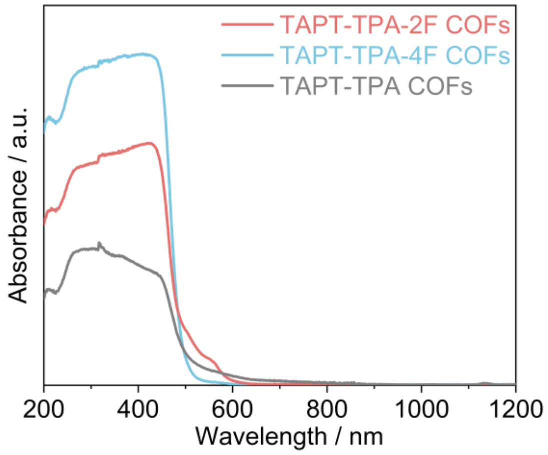

Supplementary Fig. 45 | UV–Vis absorption spectra. UV–Vis absorption spectra of TAPT-TPA-2F, TAPT-TPA-4F and TAPT-TPA COFs.

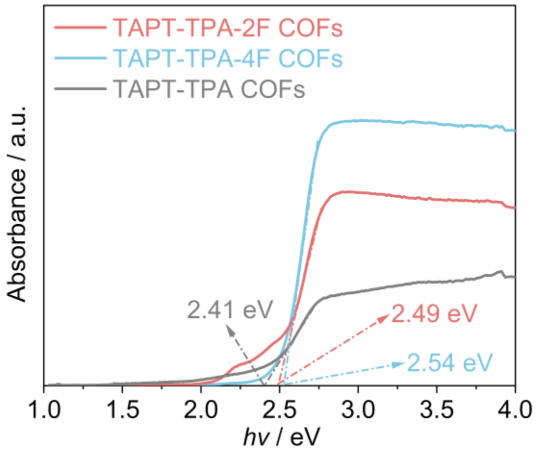

Supplementary Fig. 46 | Optical band gap analysis. Optical band gaps of TAPT-TPA-2F, TAPT-TPA-4F and TAPT-TPA COFs determined from UV–Vis absorption data using Tauc plots.

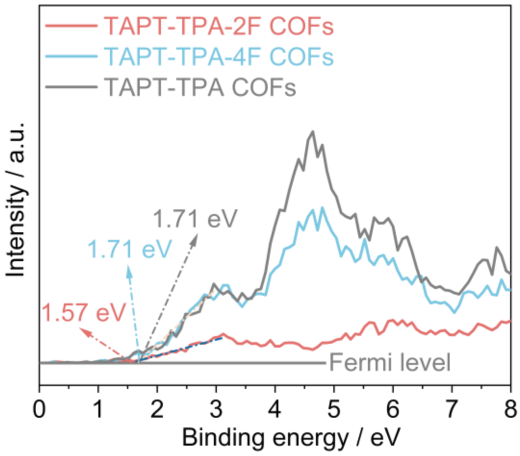

Supplementary Fig. 47 | Valence band structure. XPS valence band spectra of TAPT-TPA-2F, TAPT-TPA-4F and TAPT-TPA COFs.

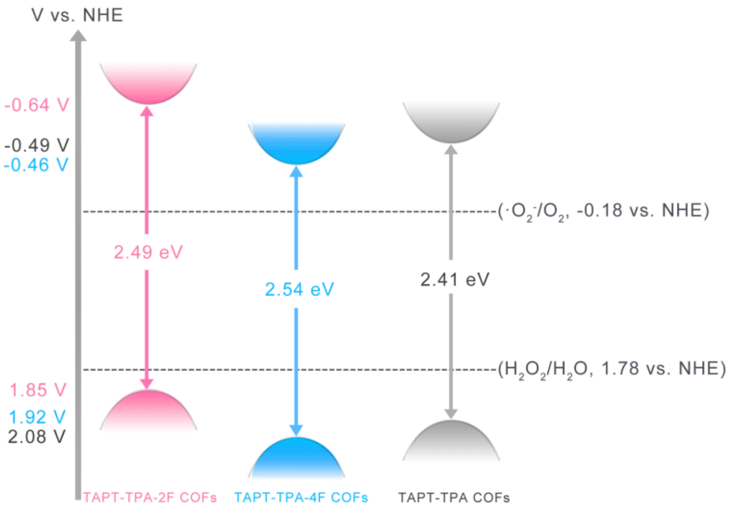

Supplementary Fig. 48 | Energy level diagram. Schematic energy level alignment of TAPT-TPA-2F, TAPT-TPA-4F, and TAPT-TPA COFs.

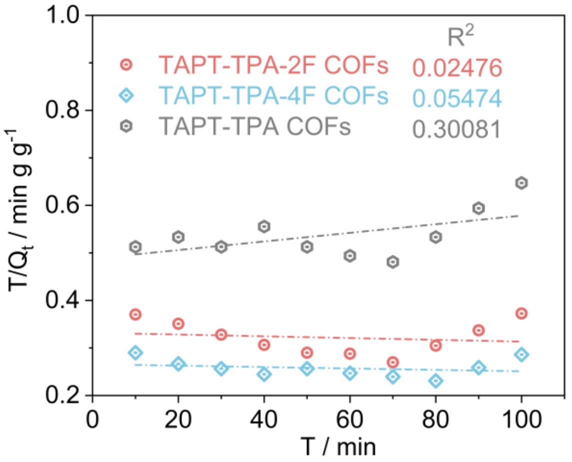

Supplementary Fig. 49 | Time-resolved U(VI) adsorption kinetics. U(VI) adsorption capacity of TAPT-TPA-2F, TAPT-TPA-4F and TAPT-TPA COFs as a function of time, with fitting curves based on the pseudo-second-order kinetic model (T = 298 K, UO_2^{2+} = 100 ppm, pH = 4).

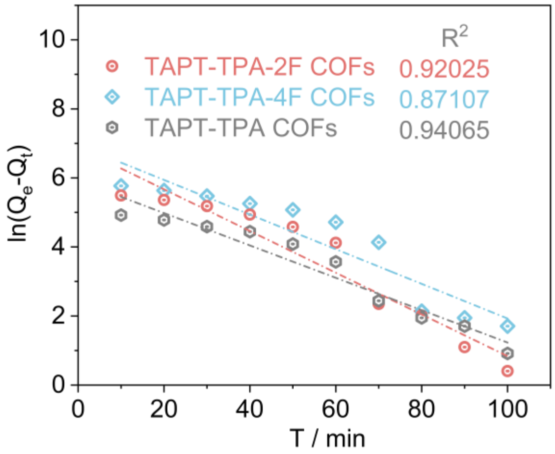

Supplementary Fig. 50 | Time-resolved U(VI) adsorption kinetics. U(VI) adsorption capacity of TAPT-TPA-2F, TAPT-TPA-4F and TAPT-TPA COFs as a function of time, with fitting curves based on the pseudo-first-order kinetic model (T = 298K, UO_2^{2+} = 100 ppm, pH = 4).

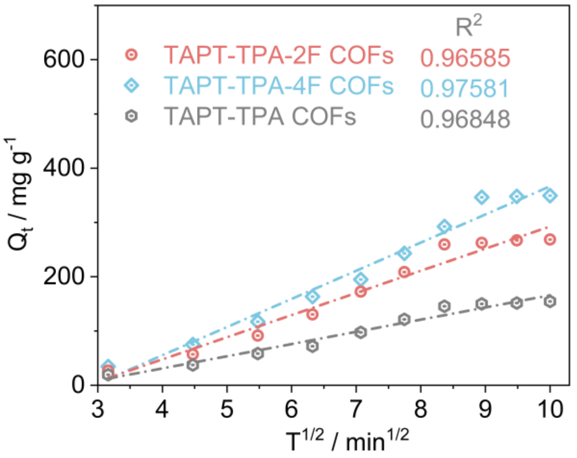

Supplementary Fig. 51 | Time-resolved U(VI) adsorption kinetics. U(VI) adsorption capacity of TAPT-TPA-2F, TAPT-TPA-4F and TAPT-TPA COFs as a function of time, with fitting curves based on the Weber-Morris model (T = 298 K, UO_2^{2+} = 100 ppm, pH = 4).

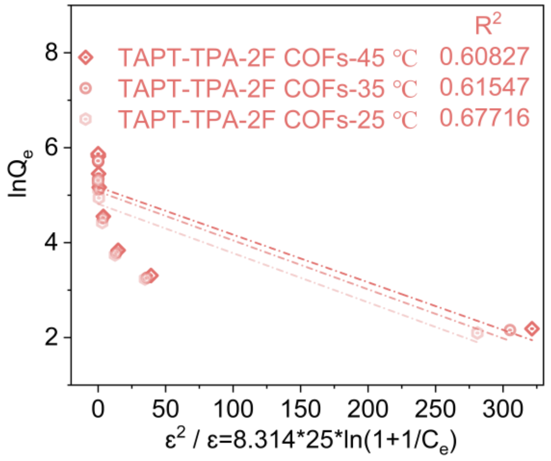

Supplementary Fig. 52 | Temperature-dependent adsorption thermodynamics of U(VI). U(VI) adsorption isotherms of TAPT-TPA-2F fitted using the Dubinin–Radushkevich (D–R) model at different temperatures (T = 298 K-318 K, pH = 4, t = 24 h).

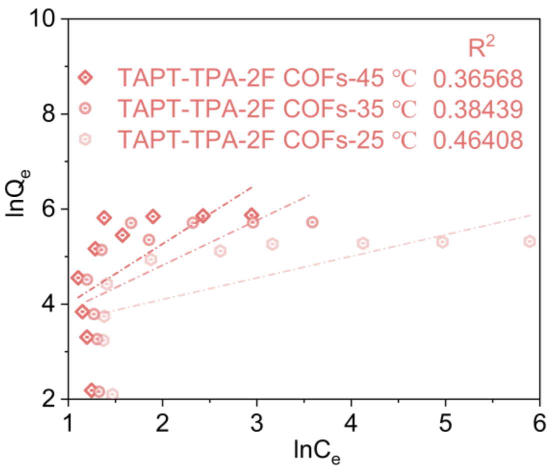

Supplementary Fig. 53 | Temperature-dependent adsorption thermodynamics of U(VI). U(VI) adsorption isotherms of TAPT-TPA-2F fitted using the Freundlich model at different temperatures (T = 298 K-318 K, pH = 4, t = 24 h).

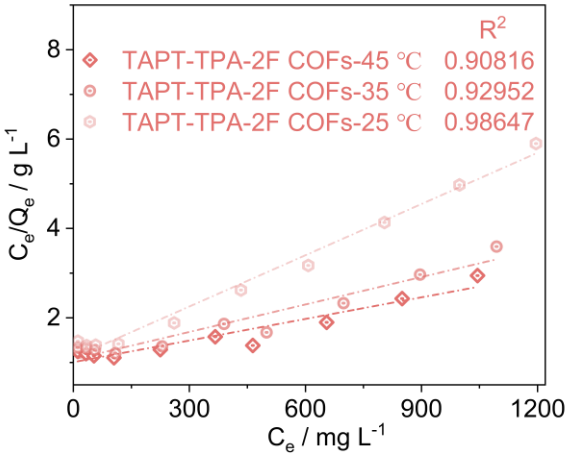

Supplementary Fig. 54 | Temperature-dependent adsorption thermodynamics of U(VI). U(VI) adsorption isotherms of TAPT-TPA-2F fitted using the Langmuir model at different temperatures (T = 298 K-318 K, pH = 4, t = 24 h).

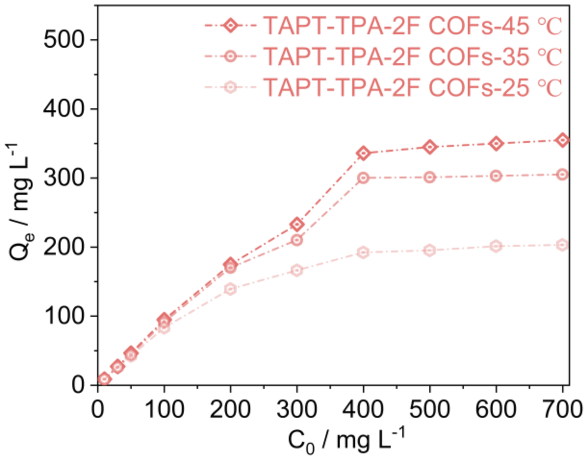

Supplementary Fig. 55 | Adsorption capacity of U(VI) at different initial concentrations. U(VI) adsorption capacities of TAPT-TPA-2F measured at varying initial uranium concentrations (T = 298 K-318 K, UO_2^{2+} = 100 ppm, pH = 4, t = 24 h).

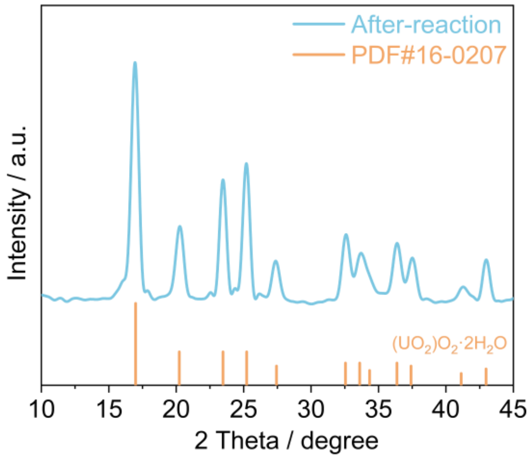

Supplementary Fig. 56 | XRD patterns of uranium-containing products after repeated photocatalytic extraction. XRD patterns of the solid products obtained after multiple photocatalytic uranium extraction cycles using TAPT-TPA-2F COFs.

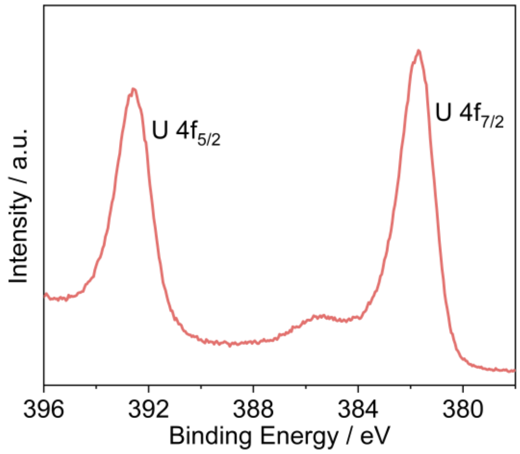

Supplementary Fig. 57 | XPS of uranium species after multiple photocatalytic cycles. High-resolution U 4f XPS spectra of the recovered products after multiple photocatalytic cycles.

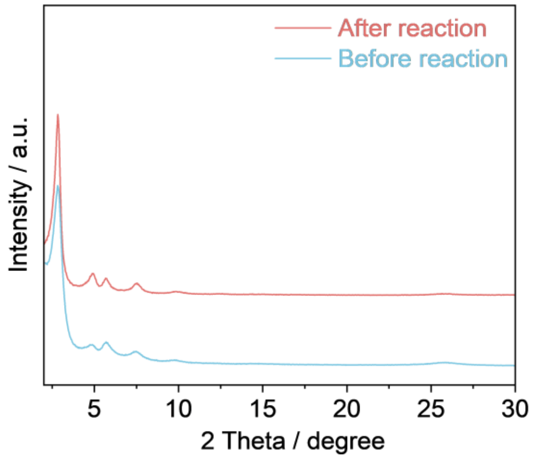

Supplementary Fig. 58 | XRD patterns before and after reaction. XRD patterns of the samples before and after the reaction.

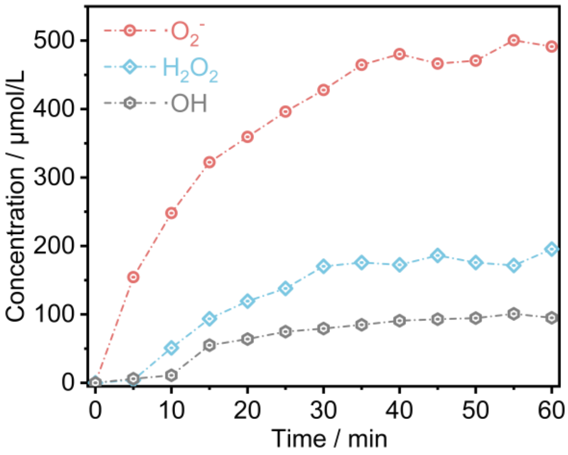

Supplementary Fig. 59 | Reactive species generated. Chemical probe method was used to identify reactive species produced by TAPT-TPA-2F COFs in solution during photocatalysis.

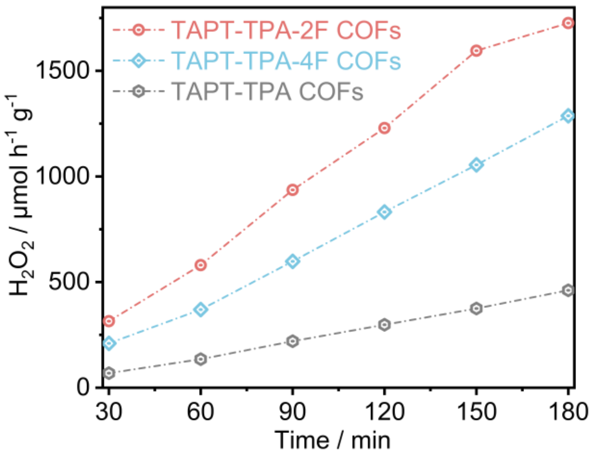

Supplementary Fig. 60 | Time-dependent photocatalytic H_2O_2 production. Photocatalytic H_2O_2 generation as a function of irradiation time using TAPT-TPA-2F, TAPT-TPA-4F and TAPT-TPA COFs.

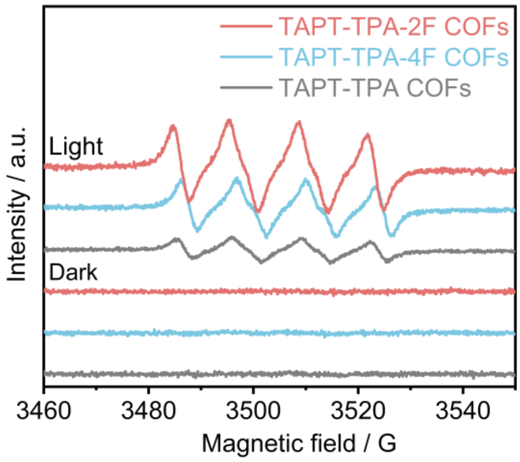

Supplementary Fig. 61 | EPR of light-induced reactive species. Electron paramagnetic resonance (EPR) spectra of TAPT-TPA-2F, TAPT-TPA-4F and TAPT-TPA COFs recorded under light and dark conditions.

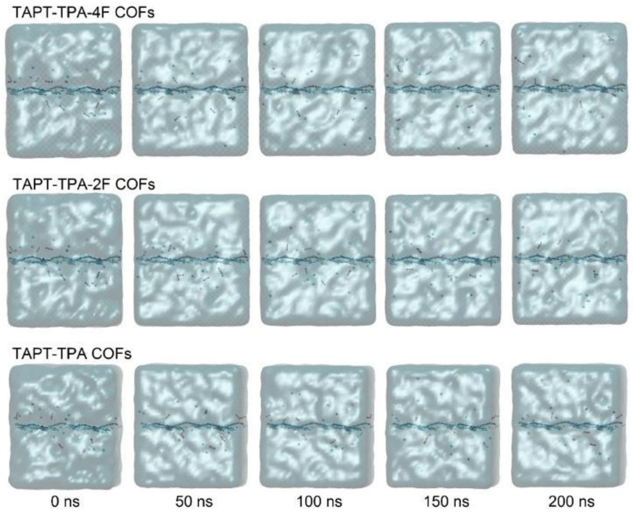

Supplementary Fig. 62 | Molecular dynamics simulation of U(VI) adsorption. Molecular dynamics simulations illustrating the uranium adsorption process on TAPT-TPA-2F, TAPT-TPA-4F and TAPT-TPA COFs (The snapshots present front views of the COF structures in aqueous solution during the adsorption process, with all solvent molecules explicitly shown).

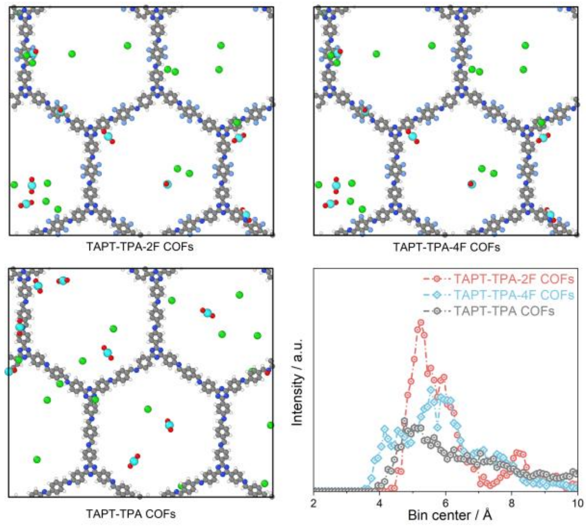

Supplementary Fig. 63 | Molecular dynamics simulation of U(VI) adsorption. Molecular dynamics simulations of uranium adsorption processes on TAPT-TPA-2F, TAPT-TPA-4F and TAPT-TPA COFs, illustrating interaction dynamics and adsorption configurations (The snapshots present top views of the systems in aqueous solution, with water molecules omitted for clarity).

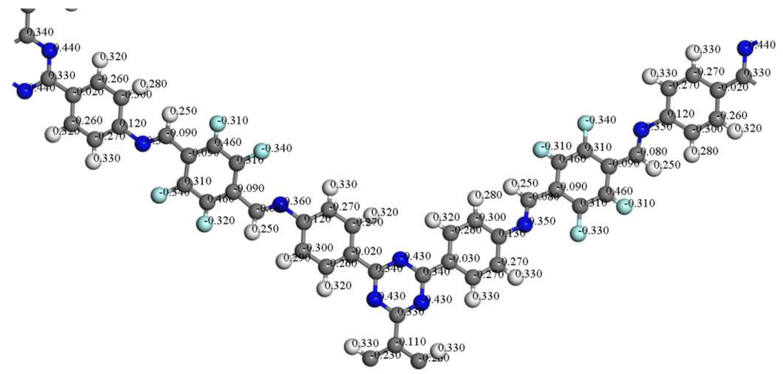

Supplementary Fig. 64 | Mulliken charge distribution. DFT-calculated Mulliken charge distribution of TAPT-TPA-4F COFs.

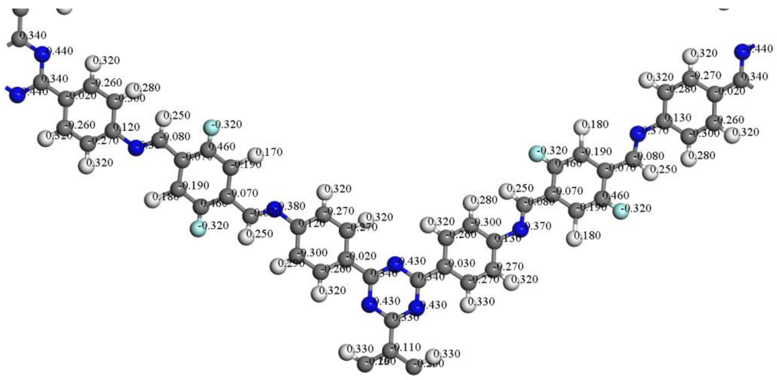

Supplementary Fig. 65 | Mulliken charge distribution. DFT-calculated Mulliken charge distribution of TAPT-TPA-2F COFs.

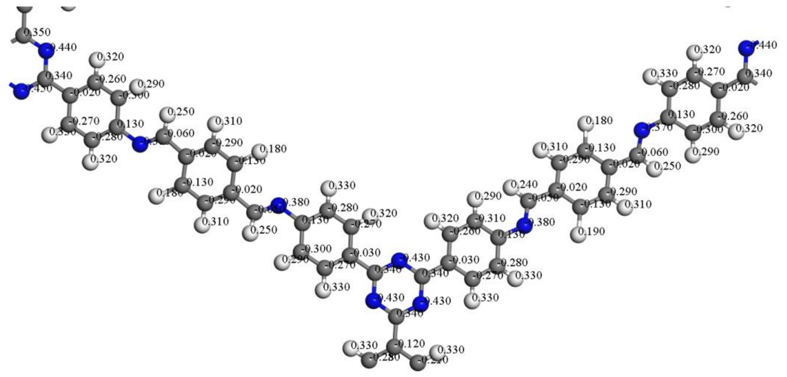

Supplementary Fig. 66 | Mulliken charge distribution. DFT-calculated Mulliken charge distribution of TAPT-TPA COFs.

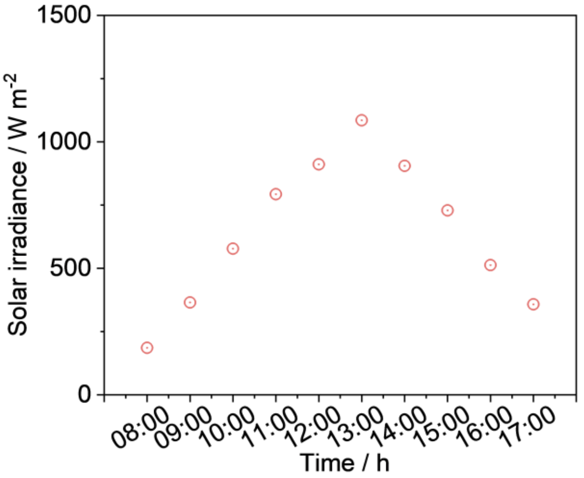

Supplementary Fig. 67 | Solar irradiance under natural sunlight. Variation of solar intensity with time during daytime under natural illumination conditions.

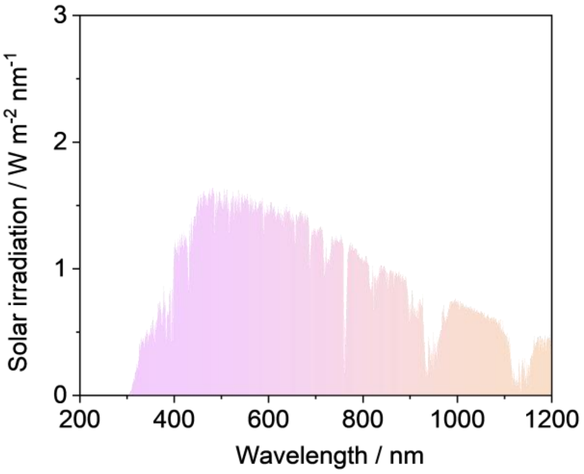

Supplementary Fig. 68 | Solar spectrum under natural illumination. Spectral distribution of solar irradiation intensity measured under natural sunlight conditions.

Supplementary Table 1. Positron annihilation lifetime parameters of TAPT-TPA-2F COFs, TAPT-TPA-4F COFs and TAPT-TPA COFs.

Sample	τ_1 (ps)	τ_2 (ps)	τ_3 (ps)
TAPT-TPA-2F	140.2	155.7	171.3
TAPT-TPA-4F	147.1	163.4	179.7
TAPT-TPA	173.8	193.2	212.5

Supplementary Table 2. Time-resolved photoluminescence lifetime parameters of TAPT-TPA-2F COFs, TAPT-TPA-4F COFs and TAPT-TPA COFs.

Sample	τ_1 (ps)	τ_2 (ps)
TAPT-TPA-2F	2.1	203.5
TAPT-TPA-4F	1.4	68.9
TAPT-TPA	1.3	39.9

Supplementary Table 3. Comparison of TAPT-TPA-2F COFs uranium removal performance with reported photocatalysts in the literature.

Photocatalyst	Time (min)	UO_2^{2+} concentration (mg/L)	Solution	Removal rate (%)	Ref.
Redox-COF1	120	270	water	97%	1
UiO-66-(COOH)	5	2	water	65%	2
TFPT-BTAN-AO	45	19.9	water	98%	3
MnO_x/UiO-66/$Ti_3C_2T_x$	120	8	water	98.4%	4
Ni-SCN@G	110	150	water	99.8%	5
PEI/PSS	200	10	water	85%	6
PyN-DAB	240	28.7	water	98%	7
$SrBi_2Nb_2O_9$	120	20	water	98%	8
Boronic-COF-1	360	100	water	92.9%	9
This work	130	100	water	99.9%	

Supplementary Table 4. Comparison of SCC for TAPT-TPA-2F COFs with the reported photocatalysts in the literature.

Photocatalyst	SCC (%)	Ref.
RF523	0.5%	10
CTF-BDDBN	0.14%	11
Sb-SAPC15	0.61%	12
RF/P3HT	1%	13
CHFs	0.78%	14
COF-TfpBpy	0.57%	15
PDT/CDT	0.55%	16
$BiVO_4$ system	0.73%	17
S_v-ZIS	0.81%	18
MSAPs-PuCN	0.82%	19
TAPT－FTPB COFs	1.22%	20
TP-DPBD3O-COF	0.91%	21
PI-BD-TPB	0.92%	22
JUC-675	1.09%	23
This work	1.52%	

Supplementary Table 5. The specific parameters of kinetics models.

**Models**	**Adsorbents**	***Q_e* cal (mg g^{-1})**	***Q_e* exp (mg g^{-1})**	**k**	**R^2**
**pseudo-first-order**	TAPT-TPA-2F	970.10	269.8	0.06042	0.92025
	TAPT-TPA-4F	1037.58	355.1	0.05017	0.8711
	TAPT-TPA	372.07	157.0	0.04688	0.94065
**pseudo-second-order**	TAPT-TPA-2F	--	269.8	1.854×10^{-4}	0.0248
	TAPT-TPA-4F	--	355.1	1.473×10^{-4}	0.0547
	TAPT-TPA	--	157.0	9.061×10^{-4}	0.3008

Supplementary Table 6. The specific parameters of isotherm models.

Models	Adsorbents	Temperature (K)	Q_m (mg g^{-1})	K	R^2
**Langmuire**	TAPT-TPA-2F	298	261.8	3.82×10^{-3}	0.98647
		308	487.8	2.05×10^{-3}	0.92952
		318	621.1	1.61×10^{-3}	0.90816
**Freundlich**	TAPT-TPA-2F	298	--	0.45399	0.46408
		308	--	0.94569	0.38439
		318	--	1.25937	0.36568
**Dubinin-Radushkevich**	TAPT-TPA-2F	298	176.17	0.01037	0.67716
		308	159.97	0.01032	0.61547
		318	176.17	0.01003	0.60827

References

1 Li, Y. *et al.* Redox-Active Two-dimensional covalent organic frameworks (COFs) for selective reductive separation of valence-variable, redox-sensitive and long-lived radionuclides. *Angew. Chem. Int. Ed.* **59**, 4168 (2020).

2 Wang, X. *et al.* In vivo uranium sequestration using a nanoscale metal-organic framework. *Angew. Chem. Int. Ed.* **60**, 1646 (2021).

3 Cui, W. R. *et al.* Regenerable and stable sp^2 carbon-conjugated covalent organic frameworks for selective detection and extraction of uranium. *Nat. Commun.* **11**, 436 (2020).

4 Yu, K. *et al.* Semiconducting metal–organic frameworks decorated with spatially separated dual cocatalysts for efficient uranium(VI) photoreduction. *Adv. Funct. Mater.* **32**, 2200315 (2022).

5 Wang, W. *et al.* Ni-single-atom mediated 2D heterostructures for highly efficient uranyl photoreduction. *Adv. Funct. Mater.* **33**, 2302913 (2023).

6 Feng, K. *et al.* Self-solidifying active droplets showing memory-induced chirality. *Adv. Sci.* **10**, 2300866 (2023).

7 Niu, C. P. *et al.* Synthesis of propenone-linked covalent organic frameworks via Claisen-Schmidt reaction for photocatalytic removal of uranium. *Nat. Commun.* **14**, 4420 (2023).

8 Guo, R. *et al.* Internal electric field-induced high-efficiency piezo-photocatalytic performance in bimetal-regulated layered perovskite $SrBi_2Nb_2O_9$. *Adv. Funct. Mater.* **34**, 2408838 (2024).

9 Zhang, Q. *et al.* Fabricating boron-functionalized covalent organic framework with remarkable potential in handling cationic, anionic, and gaseous nuclear wastes. *Adv. Funct. Mater.* **34**, 2401775 (2024).

10 Shiraishi, Y. *et al.* Resorcinol-formaldehyde resins as metal-free semiconductor photocatalysts for solar-to-hydrogen peroxide energy conversion. *Nat. Mater.* **18**, 985 (2019).

11 Chen, L. *et al.* Acetylene and diacetylene functionalized covalent triazine frameworks as metal-free photocatalysts for hydrogen peroxide production: A new two-electron water oxidation pathway. *Adv. Mater.* **32**, 1904433 (2020).

12 Teng, Z. *et al.* Atomically dispersed antimony on carbon nitride for the artificial photosynthesis of hydrogen peroxide. *Nat. Catal.* **4**, 374 (2021).

13 Shiraishi, Y. *et al.* Polythiophene-doped resorcinol-formaldehyde resin photocatalysts for solar-to-hydrogen peroxide energy conversion. *J. Am. Chem. Soc.* **143**, 12590 (2021).

14 Cheng, H. *et al.* Rational design of covalent heptazine frameworks with spatially separated redox centers for high-efficiency photocatalytic hydrogen peroxide production. *Adv. Mater.* **34**, 2107480 (2022).

15 Kou, M. *et al.* Molecularly engineered covalent organic frameworks for hydrogen peroxide photosynthesis. *Angew. Chem. Int. Ed.* **61**, 202200413 (2022).

16 Ma, J. *et al.* Extended conjugation tuning carbon nitride for non-sacrificial H_2O_2 photosynthesis and hypoxic tumor therapy. *Angew. Chem. Int. Ed.* **61**, 202210856 (2022).

17 Liu, T. *et al.* A general interfacial-energetics-tuning strategy for enhanced artificial photosynthesis. *Nat. Commun.* **13**, 7783 (2022).

18 Peng, H. *et al.* Defective $ZnIn_2S_4$ nanosheets for visible-light and sacrificial-agent-free H_2O_2 photosynthesis via O_2/H_2O redox. *J. Am. Chem. Soc.* **145**, 27757 (2023).

19 Zhang, X. *et al.* Developing Ni single-atom sites in carbon nitride for efficient photocatalytic H_2O_2 production. *Nat. Commun.* **14**, 7115 (2023).

20 Liu, Y. *et al.* Enhanced hydrogen peroxide photosynthesis in covalent organic frameworks through induced asymmetric electron distribution. *Nat. Synth.* **4**, 134 (2024).

21 Chen, Y. *et al.* Hierarchical assembly of donor–acceptor covalent organic frameworks for photosynthesis of hydrogen peroxide from water and air. *Nat. Synth.* **3**, 998 (2024).

22 Chi, W. *et al.* A photocatalytic redox cycle over a polyimide catalyst drives efficient solar-to-H_2O_2 conversion. *Nat. Commun.* **15**, 5316 (2024).

23 Liu, J. C. *et al.* Constructing donor-acceptor covalent organic frameworks for highly efficient H_2O_2 photosynthesis coupled with oxidative organic transformations. *Angew. Chem. Int. Ed.* **64**, 202416240 (2024).
